# Metformin Promotes the Protection of Mice Infected With *Plasmodium yoelii* Independently of γδ T Cell Expansion

**DOI:** 10.3389/fimmu.2018.02942

**Published:** 2018-12-13

**Authors:** Mana Miyakoda, Ganchimeg Bayarsaikhan, Daisuke Kimura, Masoud Akbari, Heiichiro Udono, Katsuyuki Yui

**Affiliations:** ^1^Division of Immunology, Department of Molecular Microbiology and Immunology, Graduate School of Biomedical Sciences, Nagasaki University, Nagasaki, Japan; ^2^Research and Education Center for Drug Fostering and Evolution, School of Pharmaceutical Sciences, Nagasaki University, Nagasaki, Japan; ^3^Department of Health, Sports, and Nutrition, Faculty of Health and Welfare, Kobe Women's University, Kobe, Japan; ^4^Department of Immunology, Graduate School of Medicine, Dentistry and Pharmaceutical Sciences, Okayama University, Okayama, Japan; ^5^Graduate School of Tropical Medicine and Global Health, Nagasaki University, Nagasaki, Japan

**Keywords:** malaria, γδ T cell, clonal expansion, protection, metformin, metabolism

## Abstract

Adaptive immune responses are critical for protection against infection with *Plasmodium* parasites. The metabolic state dramatically changes in T cells during activation and the memory phase. Recent findings suggest that metformin, a medication for treating type-II diabetes, enhances T-cell immune responses by modulating lymphocyte metabolism. In this study, we investigated whether metformin could enhance anti-malaria immunity. Mice were infected with *Plasmodium yoelii* and administered metformin. Levels of parasitemia were reduced in treated mice compared with those in untreated mice, starting at ~2 weeks post-infection. The number of γδ T cells dramatically increased in the spleens of treated mice compared with that in untreated mice during the later phase of infection, while that of αβ T cells did not. The proportions of Vγ1^+^ and Vγ2^+^ γδ T cells increased, suggesting that activated cells were selectively expanded. However, these γδ T cells expressed inhibitory receptors and had severe defects in cytokine production, suggesting that they were in a state of exhaustion. Metformin was unable to rescue the cells from exhaustion at this stage. Depletion of γδ T cells with antibody treatment did not affect the reduction of parasitemia in metformin-treated mice, suggesting that the effect of metformin on the reduction of parasitemia was independent of γδ T cells.

## Introduction

Malaria is caused by infection with *Plasmodium* parasites and is one of the most serious infectious diseases in the world. In endemic areas of tropical and subtropical countries, more than two million people suffer from malaria and ~445,000 people died from the disease in 2016, according to a World Health Organization (WHO) malaria report (1). Strains of *Plasmodium falciparum* resistant to drugs, including artemisinin, are emerging and there is an immediate need for the development of effective vaccines. However, repeated infections and a prolonged amount of time are required for people living in endemic countries to gain natural resistance to malaria, and the memory response to *Plasmodium* antigens appears to be lost in the absence of repeated infections (2, 3). It is important to define and understand the underlying mechanisms involved in the formation and maintenance of adaptive immune responses against *Plasmodium* infections to devise novel strategies for developing a malaria vaccine and to improve its effectiveness.

While antibody and CD4^+^ T-cell responses are the primary effector mechanisms of protective immunity against blood-stage infection with *Plasmodium* parasites, several studies indicate that γδ T cells also participate in the immune response. Infection of humans with *P. falciparum* is associated with increased numbers of polyclonal γδ T cells in the peripheral blood (4, 5). In particular, γδ T cells expressing Vγ9 and Vδ2 are activated by the recognition of phosphorylated molecules of *P. falciparum*, resulting in cell proliferation and IFN-γ production (6, 7). Human γδ T cells inhibit replication and kill *P. falciparum* merozoites in a cell–cell contact-dependent manner, suggesting a protective role of γδ T cells against *Plasmodium* parasites (8). Another study showed that the reduction of Vδ2^+^ γδ T cells, which respond to *P. falciparum*, in children repeatedly exposed to *Plasmodium* infection was associated with a reduced likelihood of symptoms upon subsequent infection with *P. falciparum*, suggesting a pathogenic role of human γδ T cells (9). In rodent malaria models, splenic γδ T cells increased 10-fold or more in C57BL/6 mice after infection with *P. chabaudi* and *P. yoelii*, while the increase of αβ T cells was limited (10–12). Unlike αβ T cells that expand during the early phase of infection, γδ T cell expansion occurs during the later period of infection (13–16). An increased abundance of γδ T cells is seen in β2-microglobulin knock-out mice, suggesting that the recognition of MHC class I is not required for the expansion of γδ T cells (11). γδ T cells produce cytokines such as IFN-γ and macrophage colony-stimulating factor (M-CSF) and have protective roles against *Plasmodium* infection (15, 16). Depletion of γδ T cells using a monoclonal antibody (mAb) resulted in persistent infection with the non-lethal *P. berghei* XAT strain, which is normally eliminated by the protective immune response (17). In this model of *P. berghei* XAT infection, γδ T cells expressed both CD40 ligand and interferon (IFN)-γ during the early phase of infection and enhanced the function of dendritic cells, thereby promoting protective immunity against parasites (15).

Recent studies revealed metabolic changes in T cells after their activation and during the generation of memory. Activated T cells switch the main pathway of adenosine triphosphate (ATP) generation from oxidative phosphorylation to glycolysis, which enables the generation of substrates required for synthesizing macromolecules such as nucleotides, proteins, and lipids, which promote rapid proliferation and effector function (18, 19). Metabolism in T cells is regulated by T-cell receptor (TCR) and cytokine-receptor signaling pathways involving Myc, hypoxia-inducible factor (HIF)-1a, and mammalian target of rapamycin (mTOR), which are crucial for regulating T cell activation and differentiation, and increasing or decreasing the metabolic output of cells in response to ligand stimulation (19). Adenosine monophosphate (AMP)-activated protein kinase (AMPK) senses the intracellular AMP/ATP ratio and induces a metabolic switch to promote ATP conservation by enhancing glucose uptake, fatty acid oxidation, mitochondrial biogenesis, and oxidative metabolism.

Metformin is widely used as an oral agent to treat patients with type-2 diabetes (20). Metformin is a derivative of the biguanide drugs, which were originally discovered as an antimalarial agent (21, 22). The antimalarial activities of the biguanide drugs were initially attributed to inhibition of the dihydrofolate reductase enzyme of the parasite, although additional mechanisms were subsequently proposed (23). Evidence suggests that the human mitochondrial respiratory-chain complex 1 is the target of metformin activity and that metformin binding to this target induces a drop in cellular ATP concentrations and increases the AMP:ATP ratio, resulting in AMPK activation (24). AMPK promotes oxidation of substrates in the mitochondria, thereby limiting the glycolytic capacity of cells (25). Recent studies suggest that metformin affects immune responses in mouse models of autoimmune disease and cancer. In a model of experimental autoimmune encephalomyelitis, treating mice with metformin resulted in reduced production of proinflammatory cytokines by T cells and slowed disease progression (26). In a cancer model, metformin enhanced the generation of memory CD8^+^ T cells and helped to improve resistance against cancer (27). Metformin protected CD8^+^ tumor-infiltrating T cells (TILs) from apoptosis and exhaustion, improved the function of TILs in producing multiple cytokines, and helped the shift from central memory to effector-memory T cell phenotypes (28). In the CD4^+^ T cell compartment, metformin treatment increased the production of regulatory T cells (Tregs) (29). However, in the tumor microenvironment, metformin reduced tumor-infiltrating Tregs and the expression of effector molecules that act on tumor-infiltrating Tregs, which allowed the enhancement of sustained anti-tumor immunity (30). Taken together, these results suggest that metformin acts on T cells in various manners, depending on the T cell subset and on the environmental context in which they are activated.

In this study, we investigated the effect of metformin on the immune response against blood-stage infection with rodent *Plasmodium* parasites. We used *P. yoelii* strains, since these parasites are often used for studies of the regulation of T cell immune responses during the blood-stage of *Plasmodium* infection (31, 32). As expected, metformin inhibited parasitemia levels in mice infected with *Plasmodium* parasites. Unexpectedly, however, metformin treatment enhanced the increase of γδT cells during the later phase of infection with *P. yoelii* 17XNL, but not that of αβT cells. However, parasite clearance was not directly linked to the increase in γδT cells.

## Materials and Methods

### Mice, Parasites, and Metformin

C57BL/6 (B6) mice were purchased from Japan SLC (Hamamatsu, Japan). Mice were maintained in the Laboratory Animal Center for Animal Research at Nagasaki University, and female mice were used at of 8–14 weeks of age. For infection experiments, a frozen stock of *P. yoelii* 17XL and *P. yoelii* 17XNL was thawed, washed, and inoculated intraperitoneally into B6 mice. When the parasitemia level reached 5–20%, the experimental mice were intraperitoneally infected with 10^4^ red blood cells infected with *P. yoelii* 17XNL or *P. yoelii* 17XL in 500 μL PBS. Parasitemia was monitored by microscopic examination of standard blood smears. For storage, infected blood samples were mixed with the same volume of Dulbecco's modified Eagle's medium containing 20% dimethyl sulfoxide and 10% fetal calf serum, and frozen at −80°C or in liquid nitrogen. For metformin treatment, mice were provided *ad libitum* with water containing 5 mg/ml metformin (Nakarai Tesque, Kyoto, Japan or Tokyo Chemical Industry, Tokyo, Japan) starting on the day of the infection, unless otherwise indicated. To deplete γδ T cells *in vivo*, the mice were inoculated intraperitoneally with the anti-TCRγδ mAb GL3 (500 μg/mouse) on day −1, 0, or 1 relative to infection and then twice a week after infection, as reported previously (15). A hybridoma cell line producing anti-TCRγδ mAb (GL3) (33) was provided by Dr. F. Kobayashi (Kyorin University, Tokyo, Japan). Cells were cultured using a BD CELLine Flask (BD Biosciences), and mAb was purified from the culture supernatant using HiTrap Protein G columns (GE Health Care, Pittsburg, PA, USA). The animal experiments reported herein were conducted in accordance with the recommendations of the guidelines for Animal Experimentation, Nagasaki University. The protocol was approved by the Institutional Animal Care and Use Committee of Nagasaki University.

### Flow Cytometry

Single cell suspensions of the spleen, inguinal lymph nodes, thymus, and bone marrow were pre-incubated with anti-CD16/CD32 mAb (2.4G2) for 10 min and then stained for 30 min with phycoerythrin (PE)-cyanine 7 (Cy7)-, fluorescein isothiocyanate (FITC)-, or BV510-conjugated anti-CD3 (145-2C11); FITC-conjugated anti-TCRβ (H57–597); PE- or allophycocyanin (APC)-conjugated anti-TCRγδ (GL3); APC-Cy7-conjugated anti-CD8 (53–6.7); BV711-conjugated anti-CD4 (RM4-5); PE-conjugated anti-TCR Vγ1(2.11); PE-conjugated anti-TCR Vγ2 (UC3-10A6); APC-conjugated anti-TCR Vγ3 (536); PE-conjugated anti-TCR Vδ4 (GL2); PE-conjugated anti-TCR Vδ6.3/2 (8F4H7B7); FITC-conjugated anti-B220 (RA3-6B2); APC-conjugated anti-CD27 (LG.3A10); FITC-conjugated anti-CD44 (IM7); PE-conjugated anti-CD69 (H1.2F3); FITC-conjugated anti-CD25 (PC61); APC-conjugated anti-TIM-3 (RMT-23); APC-conjugated anti-LAG-3 (C9B7W); APC-conjugated anti-PD-1 (RMP1-30); APC-conjugated anti-KLRG1 (2F1/KLRG1) mAbs; or biotin-conjugated anti-CD62L mAb (MEL-14) plus PE-conjugated streptavidin. Cells were stained with PE-conjugated anti-NKG2D (CX5) and FITC-conjugated anti-FCγRIII/II (2.4G2) without an anti-CD16/CD32 mAb. The antibodies were purchased from eBioscience (San Diego, CA, USA), BD Biosciences (San Jose, CA, USA), TONBO Biosciences (San Diego, CA, USA), or BioLegend (San Diego, CA, USA). To exclude dead cells from the analysis, 7-amino-actinomycin D (7-AAD; eBioscience) was added.

Analysis of cellular metabolism was performed as described previously (34). Briefly, splenocytes were pulsed with 50 μM 2-(N-(7-nitrobenz-2-oxa-1,3-diazol-4-yl) amino)-2-deoxyglucose (2NBDG; Molecular Probes) for 30 min at 37°C and then stained for surface molecules in the presence of 20 nM MitoTracker Deep Red FM (MitoFM; Invitrogen, Carlsbad, CA, USA) for 30 min at 4°C. Samples were analyzed using a BD FACSCanto II or FACS Fortessa instrument (BD Biosciences). The data were analyzed using FlowJo software v10.2 (TreeStar, Ashland, OR). The numbers of spleen cells in each subset were determined by multiplying the total number of spleen cells by the percentage in each subset.

### Intracellular Staining

To detect phosphorylated proteins, cells were stained with an APC-conjugated anti-TCRγδ mAb, fixed using a Phosflow Kit (BD Biosciences), and stained with a FITC-conjugated anti-CD3 mAb and PE-conjugated phospho-S6 (ser235/Ser236) antibody (eBioscience), a PE-conjugated phospho-mTOR (Ser2448) antibody (eBioscience), or a phospho-AMPK alpha 1 (phosphor T183) + AMPK alpha 2 (phosphor T172) antibody (Abcam, Cambridge, MA USA) plus a PE-conjugated anti-rabbit IgG antibody. To label proliferating cells, mice were intraperitoneally administered 1.4 mg bromodeoxyuridine (BrdU; BD Biosciences) 16 h prior to analysis. After staining cell-surface molecules, splenocytes were fixed, permeabilized, incubated with DNase, and intracellularly stained with an FITC-conjugated anti-BrdU mAb (3D4) (BioLegend), as described previously (35). To analyze cell death, cells were stained with annexin V (BioLegend) in annexin V-binding buffer (10 mM HEPES, 2.5 mM CaCl_2_, 0.14 M NaCl, pH 7.5) for 30 min in accordance with the manufacturer's instructions (Sigma–Aldrich, St. Louis, MO, USA) and were then stained with 7-AAD for 10 min.

For cytokine assays, splenocytes (1 × 10^6^/well) were stimulated in 24-well plates with phorbol 12-myristate 13-acetate (PMA; 50 ng/ml) and ionomycin (1 μM) for 6 h in RPMI 1640 medium, supplemented with non-essential amino acids, glutamine, sodium pyruvate, antibiotics, 10% fetal calf serum, 2-mercaptoethanol (5 × 10^−5^ M), and monensin. After pre-incubation with the anti-CD16/CD32 antibody 2.4G2, cells were stained with APC-conjugated anti-TCRγδ and BV510-conjugated anti-CD3 mAb; fixed and permeabilized using Cytofix/Cytoperm buffer (BD Biosciences); and stained with PE-Cy7-conjugated anti-IFN-γ (XMG1.2) (BioLegend), PE-conjugated anti-tumor necrosis factor (TNF)α (MP6-XT22) (eBioscience), PE-Cy7-conjugated anti-IL-2 (JES6-5H4) (eBioscience), or PE-conjugated anti-granzyme B (NGZB) (eBioscience) mAbs and then analyzed using a BD LSRFortessa X-20 instrument (BD Biosciences).

### Immunohistochemistry

Spleen tissues were prepared for immunohistochemistry as previously described (36). Briefly, freshly frozen spleens were embedded in Tissue-Tek OCT compound (Sakura Finetek, Tokyo, Japan), cut into 5-μm-thick sections using a cryomicrotome, and fixed in acetone for 15 min at room temperature. Samples were blocked using Blocking One Histo (Nacalai, Kyoto, Japan) for 1 h in a humidified chamber at room temperature. Sections were stained overnight at 4°C with PE-conjugated anti-TCRγδ and FITC-conjugated anti-TCRβ mAbs (all from BioLegend) and mounted in DAKO fluorescent mounting medium (Agilent Tech, Santa Clara, CA, USA). Images were acquired by fluorescence microscopy (Olympus, Tokyo, Japan) and merged using ImageJ software (National Institutes of Health, Bethesda, MD, USA).

### Statistical Analysis

The data are presented in bar graphs as the mean ± the standard error of the mean (SEM). Statistical analysis was performed using GraphPad Prism (GraphPad Software). Comparison of two independent groups was performed using the unpaired *t*-test with Welch's correction. Differences between multiple groups were analyzed by one-way analysis of variance (ANOVA) with Tukey's *post-hoc* test. ^***^p < 0.001, ^**^p < 0.01, ^*^p < 0.05. ns, not significant. Survival curves were compared using a log-rank (Mantel cox) test.

## Results

### Metformin Promoted Clearance of Plasmodium Parasites

**Figure 1:**
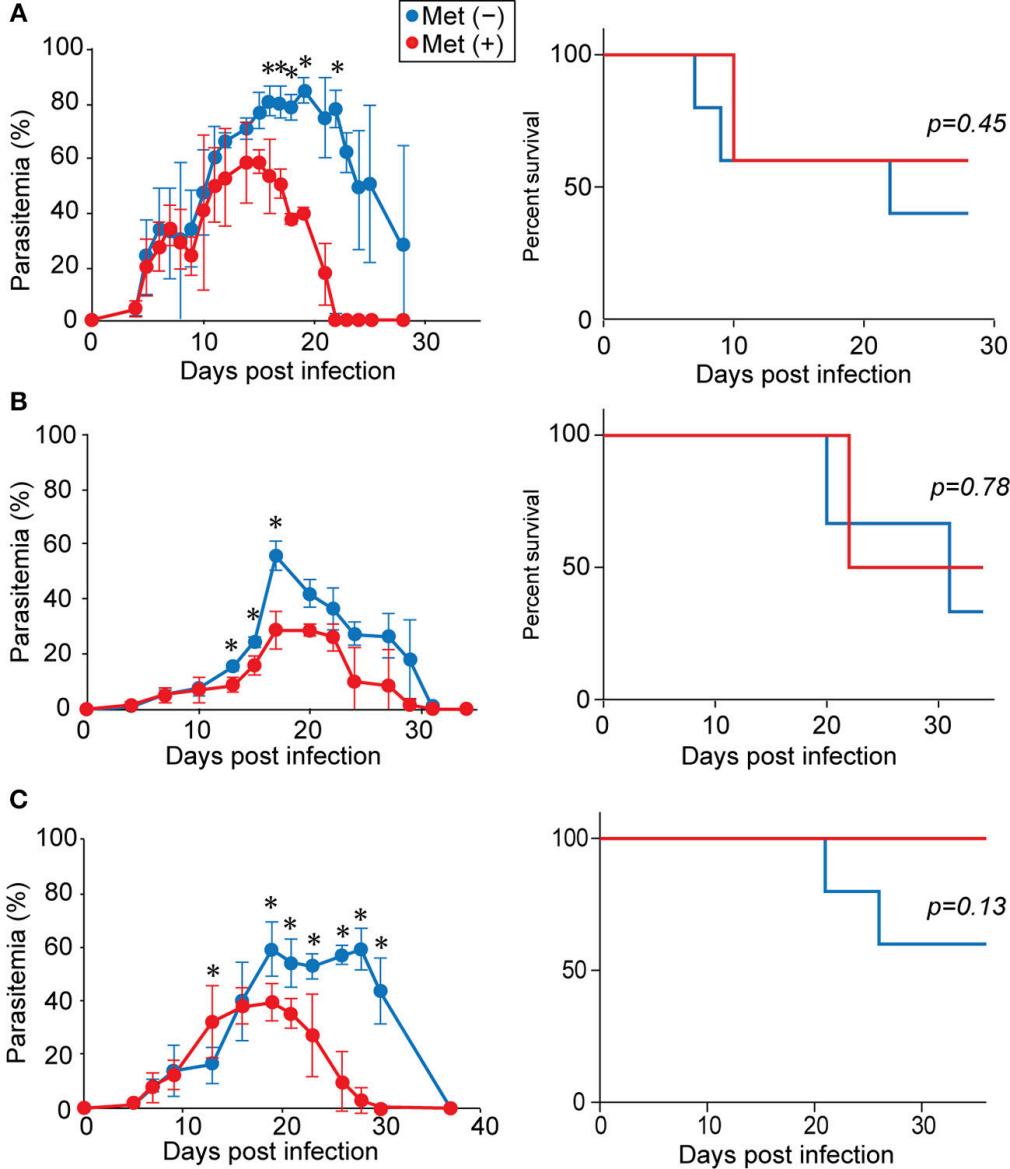
Metformin reduced parasitemia and promoted the clearance of *Plasmodium* parasites. B6 mice were infected with *P. yoelii* 17XL **(A)** or *P. yoelii* 17XNL **(B)** and received metformin (Met^+^, red line) or not (Met^−^, blue line) in their drinking water, starting from the day of infection. Alternatively, B6 mice were infected with *P. yoelii* 17XNL and either did (red line) or did not (blue line) receive metformin in their drinking water, starting at 7 d post-infection **(C)**. The levels of parasitemia were monitored every few days. The data shown represent two **(A)**, three **(B)**, or one **(C)** experiment with 5 mice/group in each experiment. Statistical significance in the differences in parasitemia was assessed between the metformin-treated and untreated mice using the unpaired *t*-test with Welch's correction (^*^p < 0.05). Survival curves were compared using a log-rank (Mantel cox) test.

To study the effect of metformin on *Plasmodium* infection, mice were infected with *P. yoelii* 17XL or *P. yoelii* 17XNL and given metformin in their drinking water, starting on the day of infection. For mice infected with *P. yoelii* 17XL (Figure 1A) or *P. yoelii* 17XNL (Figure 1B), the levels of parasitemia increased similarly in both treated and untreated groups for ~2 weeks post-infection. Lethal and non-lethal phenotypes of *P. yoelii* 17XL and *P. yoelii* 17XNL, respectively, are not always stable between experiments (37). Under our experimental conditions, some B6 mice survived *P. yoelii* 17XL infection, and *P. yoelii* 17XNL infection was lethal in some of the infected B6 mice. In *P. yoelii* 17XL-infected mice, nearly half of the metformin-treated and untreated mice died ~10 days after infection. Among the surviving mice, the metformin-treated mice showed lower parasitemia levels and cleared the parasites earlier than untreated mice. In *P. yoelii* 17XNL-infected mice, the parasitemia levels were lower after metformin treatment, while no significant difference was observed in survival between the metformin-treated and untreated mice. We used the *P. yoelii* 17XNL-infection model in further experiments, since all mice survived at least 20 days using this model. This effect of metformin on *P. yoelii* 17XNL parasitemia was also observed when the mice were given metformin starting at 7 d post infection (Figure 1C). These results suggested that metformin promoted the clearance of the *Plasmodium* parasites in mice. In further study, we continuously treated B6 mice with metformin starting at day 0 of infection with *P. yoelii* 17XNL, since the level of parasitemia was stably reduced on day 18 in this model.

### Increased γδ T Cells in Metformin-Treated Mice

**Figure 2:**
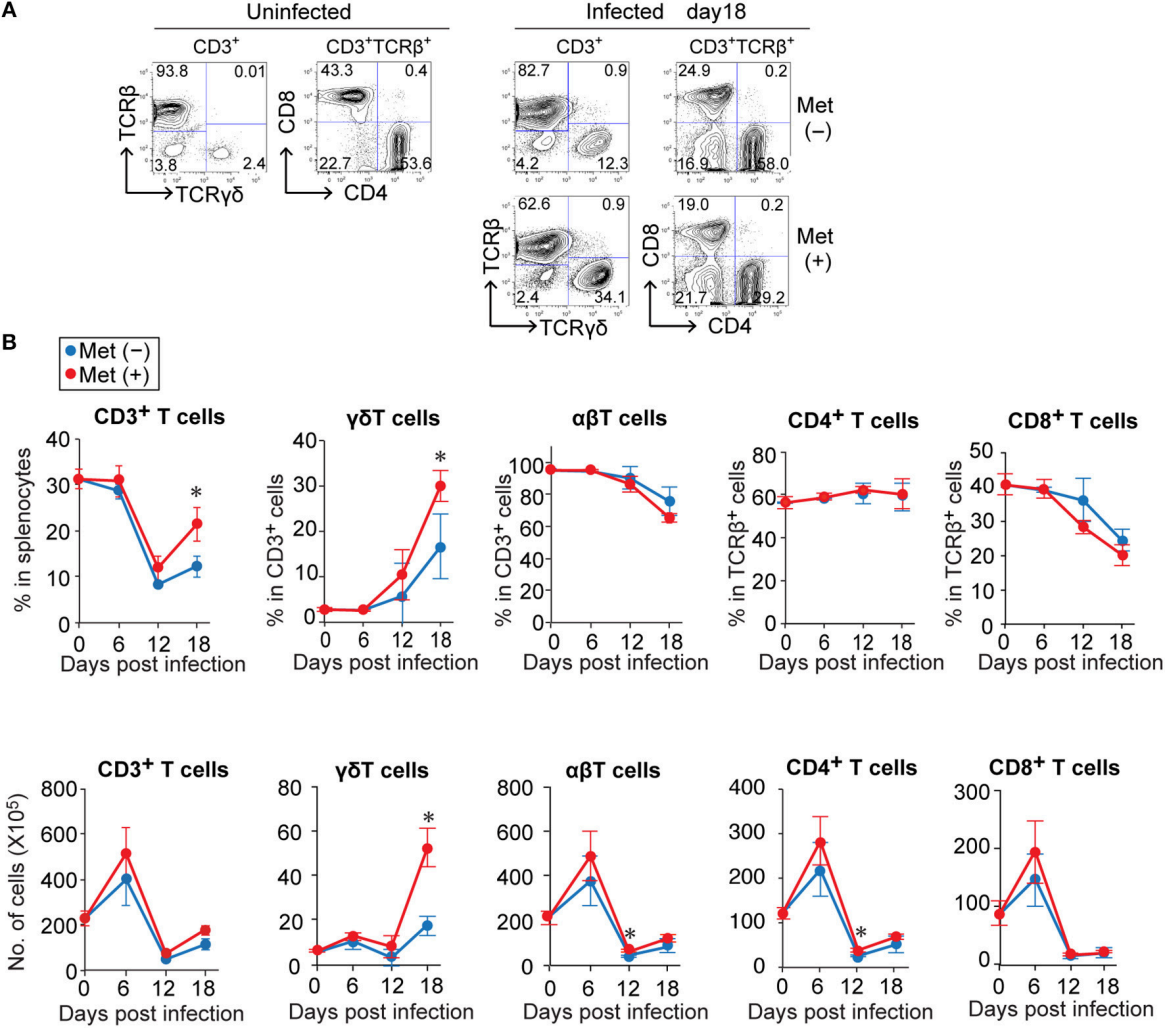
The relative increase of γδ T cells in *Plasmodium yoelii* 17XNL-infected mice during the late stage of infection was greater for metformin-treated mice. B6 mice were infected with *P. yoelii* 17XNL and given metformin (Met^+^) or not (Met^−^) in their drinking water, starting from the day of infection. Splenocytes from uninfected and infected mice were stained for CD3, TCRβ, TCRγδ, CD4, and CD8 expression before (0 d) and at 6, 12, and 18 d post-infection, and were analyzed by flow cytometry. **(A)** Representative data for CD3^+^-gated cells and CD3^+^TCRβ^+^-gated cells before (uninfected) and 18 days after infection are shown. The number in each quadrant represents the proportion of the indicated cell populations. **(B)** The proportions (upper panel) and absolute numbers (lower panel) of T cell subpopulations in the spleens of mice infected with *P. yoelii* 17XNL at 0, 6, 12, and 18 d post-infection. The numbers were calculated by multiplying the total number of splenocytes by the relative proportion of each subpopulation. Each group was comprised of three mice. The data shown represent two independent experiments with similar results. Statistical significance was assessed using the unpaired *t*-test with Welch's correction (^*^p < 0.05).

**Figure 3:**
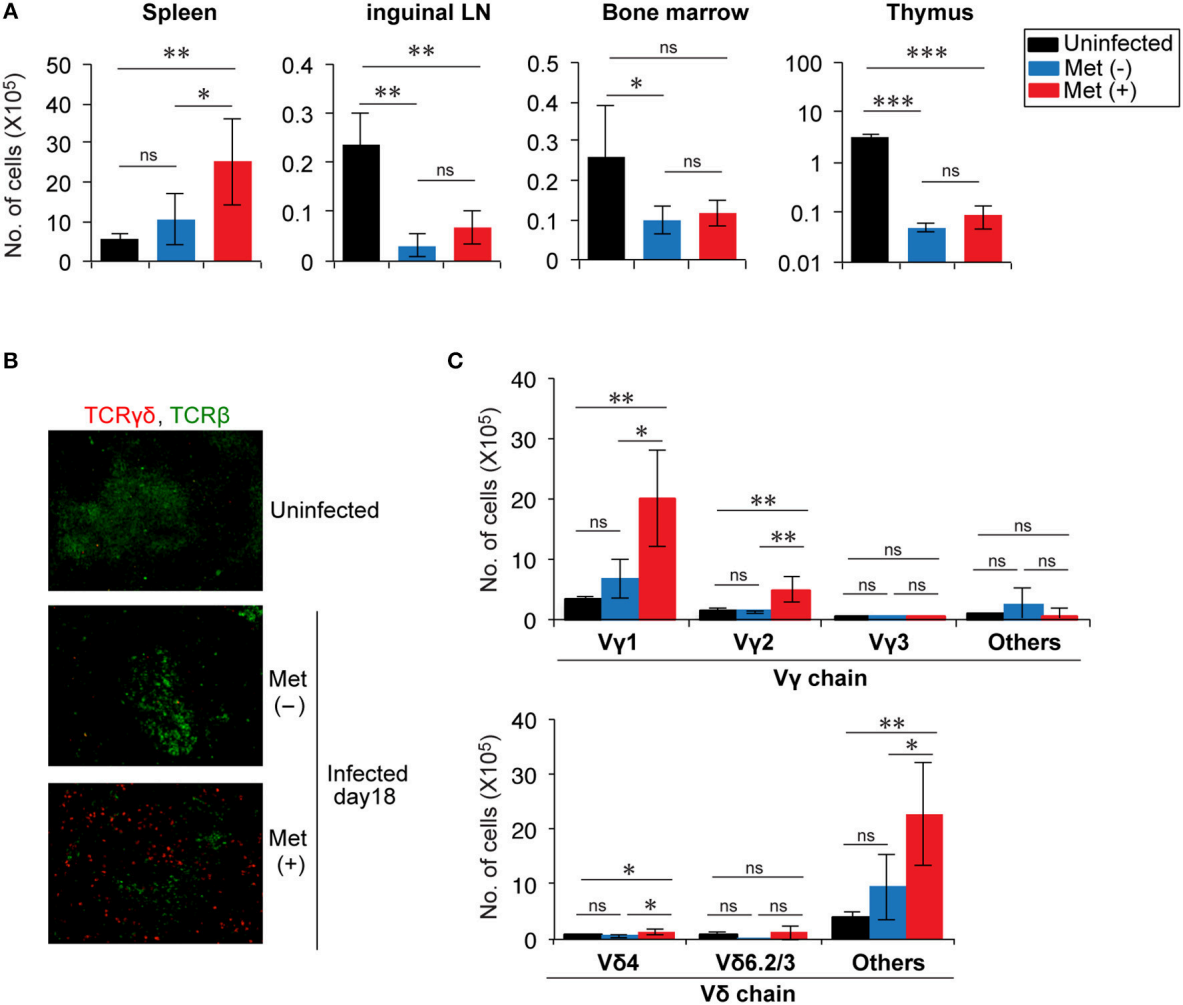
Expansion of Vγ1^+^ and Vγ2^+^ T cells in the splenic red pulp of *Plasmodium yoelii* 17XNL-infected mice treated with metformin. **(A)** B6 mice were infected with *P. yoelii* 17XNL and given metformin (Met^+^) or not (Met^−^), as indicated. At 18 d post-infection, the numbers of γδ T cells in the spleens, inguinal lymph nodes, bone marrow, and thymus from uninfected and infected mice were determined as described in the legend of Figure 2. The data shown represent two independent experiments (3–4 mice/group) with similar results. Statistical significance was assessed by one-way ANOVA with Tukey's *post-hoc* test (^*^p < 0.05, ^**^p < 0.01, ^***^p < 0.001). **(B)** Spleen sections from uninfected and *P. yoelii* 17XNL-infected mice were stained with anti-TCRγδ (red) and anti-TCRβ (green) mAbs. **(C)** At 18 d post-infection, splenocytes from infected and uninfected mice were stained for CD3, TCRγδ, and for Vγ or Vδ, and analyzed by flow cytometry. The numbers of γδ T cells expressing the particular Vγ or Vδ chains are shown. The data shown represent two independent experiments (3–4 mice/group) with similar results. Statistical significance was assessed using one-way ANOVA with Tukey's *post-hoc* test (^*^p < 0.05, ^**^p < 0.01, ns, not significant).

Since metformin was previously found to improve the function of TILs (28), we examined the T-cell populations in the spleens of mice during *P. yoelii* 17XNL-infection using flow cytometry (Figure 2, Figure S1). The number of T cells (CD3^+^ cells) increased in the first 6 days, decreased dramatically over the next 6 days, and then slightly increased during the following 6 days of infection in both metformin-treated and untreated mice (Figure 2B). This increase was not observed in mice treated with metformin alone for 18 days without *Plasmodium* infection (Figure S2). Consistent with a previous report, the proportion of γδ T cells among CD3^+^ T cells increased by 18 d post-infection (Figures 2A,B) (15). However, the increase of γδ T cells was much higher in metformin-treated mice than in un-treated mice and reached >30% of the CD3^+^ cells (Figure 2A). The number of γδ T cells in the spleen was stable during the initial 12 d of infection and increased between 12 and 18 d post-infection (Figure 2B). However, in the αβT cell fraction, both CD4^+^ and CD8^+^ T cells increased during the initial 6 d of infection and decreased thereafter (Figure 2B). No significant difference was found in the number of αβ T cells between the metformin-treated and untreated mice at 18 d post-infection. The increase in the number of γδ T cells occurred in the spleens of the metformin-treated mice, but not in the lymph nodes, bone marrow, or thymus (Figure 3A and Figure S1). No significant difference in B cell number occurred between metformin-treated and untreated mice (Figure S3A). The number of conventional dendritic cells was slightly higher in metformin-treated mice 12 days after infection with *P. yoelii* 17XNL. We did not find significant differences in serum levels of anti-parasite antibodies between metformin-treated and untreated mice, except for IgG3 levels 12 days after infection (Figure S3B).

Spleen sections were subjected to immunohistochemical analysis to determine the distribution of γδ T cells in the spleens (Figure 3B). γδ T cells were scattered in an area distinct from αβ T cells, suggesting that they were localized primarily in the red pulp of the spleen, consistent with a previous report (15). We then examined the frequencies of Vγ and Vδ usages in the increased γδ T cells (Figure 3C and Figure S1). The number of γδ T cells expressing Vγ1 or Vγ2 and those expressing Vδ chains, other than Vδ4 or Vδ6, significantly increased in metformin-treated mice. These cells showed a tendency to be increased even in metformin-untreated mice by infection with *P. yoelii* 17XNL, although the difference was not statistically significant. Therefore, we think that these γδ T cells were activated via the recognition of *Plasmodium* antigens in *P. yoelii* 17XNL-infected mice, and their expansion was enhanced by metformin treatment.

**Figure 4:**
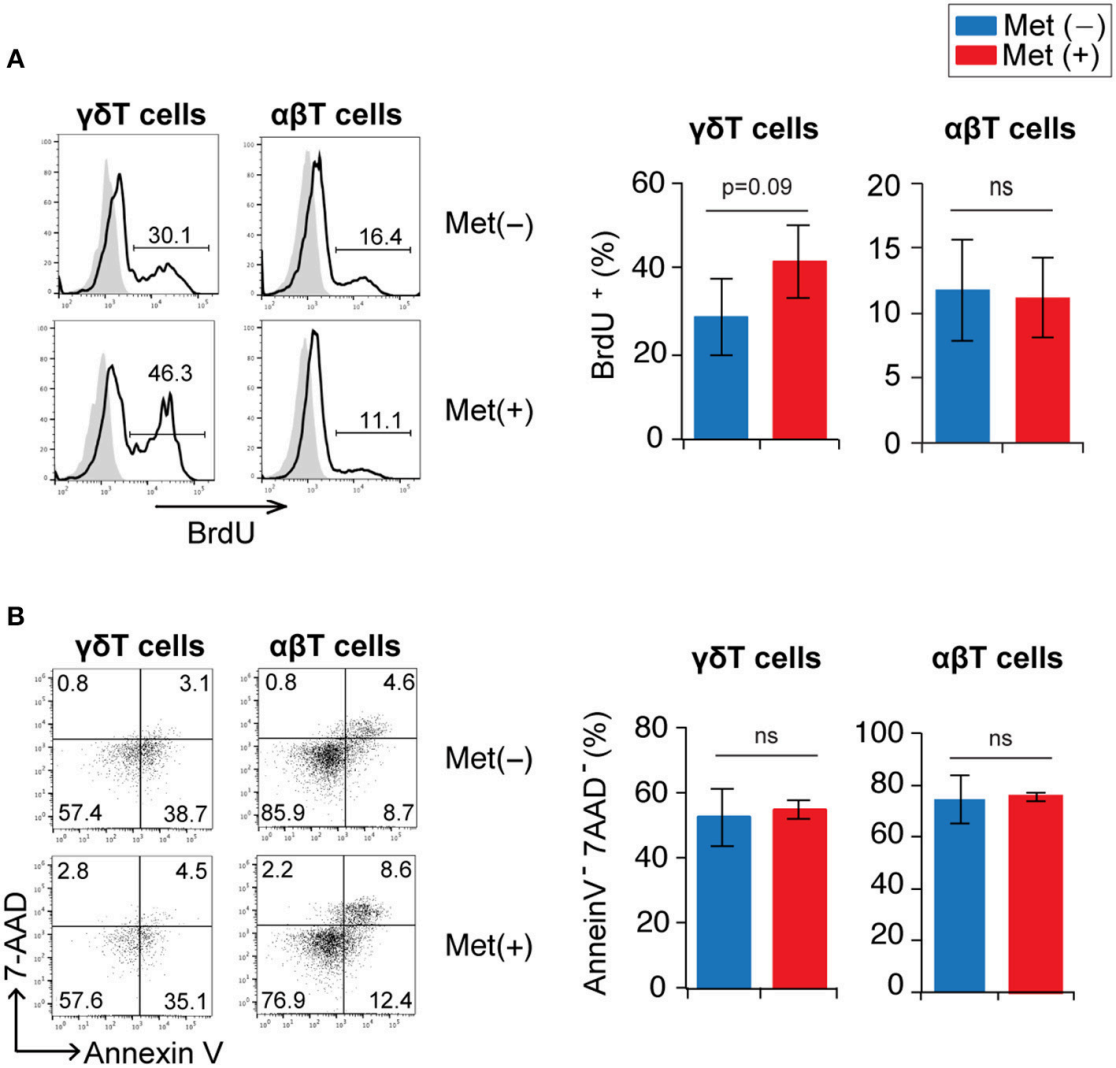
Proliferation and survival of γδ T cells in *Plasmodium yoelii* 17XNL-infected mice by metformin. B6 mice were infected with *P. yoelii* 17XNL and treated with metformin (Met^+^) or not (Met^−^), as indicated. One day prior to analysis, mice received an intraperitoneal injection of BrdU. Mice were sacrificed at 16 or 18 d post-infection (3 mice/group). **(A)** Spleen cells were surface stained with anti-CD3 and anti-TCRγδ mAbs and stained intracellularly with an anti-BrdU mAb. BrdU-staining profiles in γδ (CD3^+^TCRγδ^+^) and αβ (CD3^+^TCRγδ^−^) T cells are shown. The numbers shown in the flow cytometry histograms indicate the proportion of cells within the region of the line. The proportion of BrdU^+^ cells in the experiments were pooled for the bar graphs (*n* = 6). **(B)** Spleen cells were stained with anti-CD3 and anti-TCRγδ mAbs, annexin V, and 7-AAD. Annexin V vs. 7-AAD staining profiles in γδ (CD3^+^TCRγδ^+^) and αβ (CD3^+^TCRγδ^−^) T cells are shown. The numbers indicate the proportions of cells in each quadrant. The proportions of annexin V^−^7-AAD^−^ cells in the experiments were pooled (*n* = 6). Statistical significance was assessed using the unpaired *t*-test with Welch's correction (ns; not significant).

The increase in the number of γδ T cells induced by metformin treatment may have been due to either enhanced proliferation or to a reduction in cell death. Proliferation was evaluated using BrdU incorporation since γδ T cells in the infected mice were nearly 100% Ki67^+^ (data not shown). Although the increase was not statistically significant, the proportion of BrdU^+^ γδ T cells in metformin-treated mice tended to be higher than that in untreated control mice, while αβ T cells in the same cell preparation showed no difference (Figure 4A and Figure S1). The proportions of surviving 7AAD^−^ annexin-V^−^ γδ T cells were not different between the metformin-treated and untreated groups of mice (Figure 4B). Taken together, our findings suggest that metformin enhanced the proliferation of γδ T cells in *P. yoelii* 17XNL-infected mice.

### Activation and Function of γδT Cells in Metformin-Treated Mice

**Figure 5:**
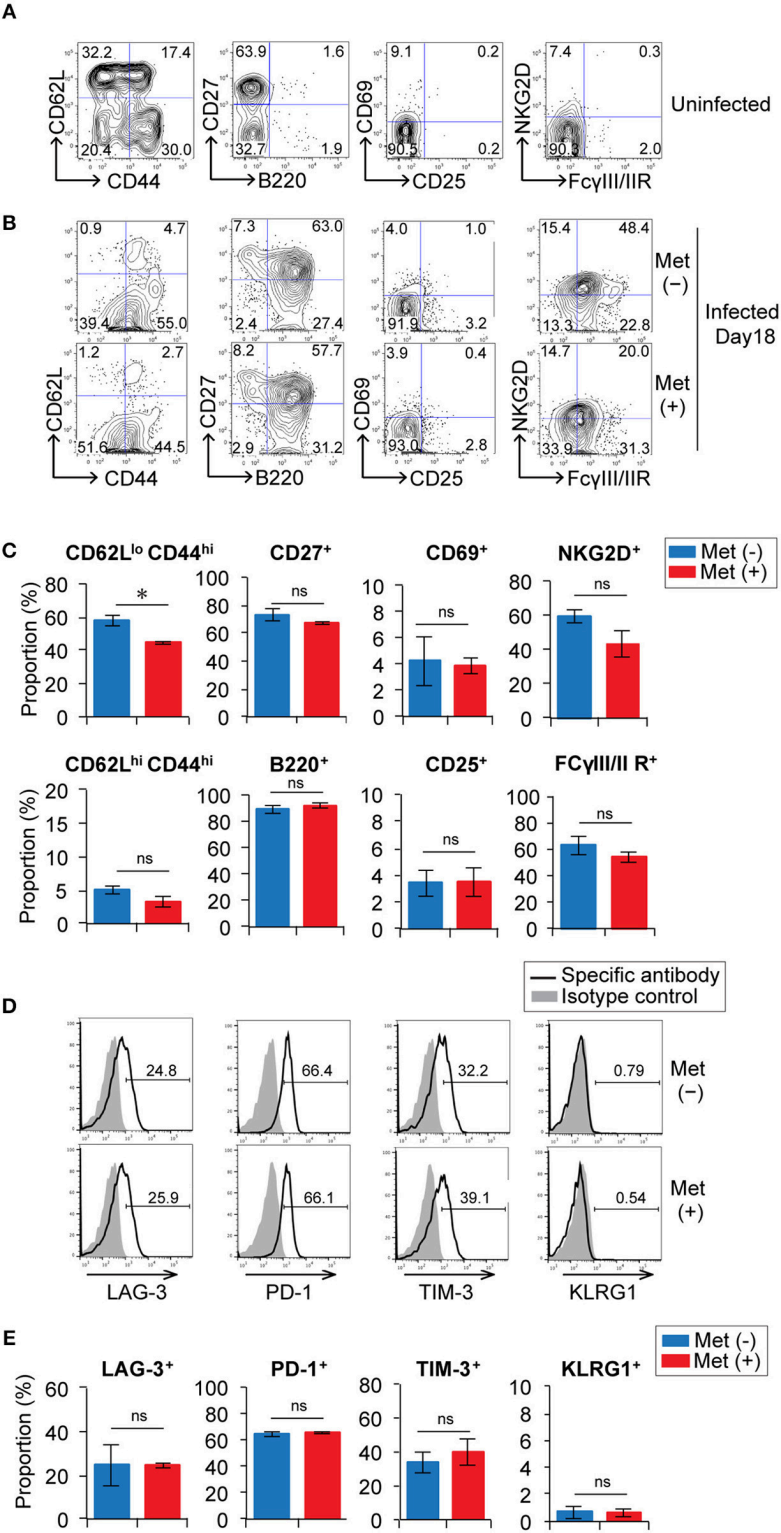
Expression of activation markers and inhibitory receptors on γδ T cells in *Plasmodium yoelii*-infected mice treated with metformin. B6 mice were mock-infected **(A)** or infected with *P. yoelii* 17XNL and treated with metformin (Met^+^) or not (Met^−^), as indicated **(B)**. At 18 d post-infection, spleen cells were stained for CD3, TCRγδ, and other cell-surface markers **(A–C)** and inhibitory receptors **(D,E)**. Representative plots of these molecules on CD3^+^ TCRγδ^+^ cells **(B,D)** and the proportions of the positive γδ T cells are shown **(C,E)**. The data shown represent two independent experiments (3 mice/group) with similar results. Statistical significance was assessed using the unpaired *t*-test with Welch's correction (^*^p < 0.05; ns, not significant).

We examined the phenotypes of γδ T cells in metformin-treated and untreated mice at 18 d post-infection with *P. yoelii* 17XNL (Figures 5A–C and Figure S1). The proportion of γδ T cells with an effector phenotype (CD62L^lo^ CD44^hi^) in metformin-treated mice was lower than that in untreated mice, while the proportion of γδ T cells with a central memory-phenotype (CD62L^hi^ CD44^hi^) was not significantly different between the metformin-treated and untreated groups of mice. In the αβ T cell compartment, however, CD62L^hi^ CD44^hi^ T cells were decreased in metformin-treated mice, while CD62L^lo^ CD44^hi^ T cells were not significantly affected (Figure S4). These changes were not observed in uninfected, metformin-treated mice (Figures S2B,C). B220 expression was upregulated on splenic γδ T cells in *P. yoelii*-infected mice, which was consistent with previously reported findings for hepatic γδ T cells (38), but B220 expression was not affected by metformin treatment. The proportions of γδ T cells expressing CD27, which is expressed on IFN-γ-producing γδ T cells (39), as well as the activation markers CD25, CD69, FCγR, and NKG2D (40–42) were not affected by metformin treatment (Figures 5A,B). The expression of inhibitory molecules including KLRG1 that possess an ITIM motif in the cytoplasmic domain and has inhibitory function in T cells (43) was also evaluated. The γδ T cells expressed similar levels of LAG-3, PD-1, TIM-3, and KLRG1 in both metformin-treated and untreated mice (Figures 5D,E). Taken together, the expression of some activation markers on γδ T cells was reduced in mice treated with metformin, while the expression of inhibitory receptors was not affected.

**Figure 6:**
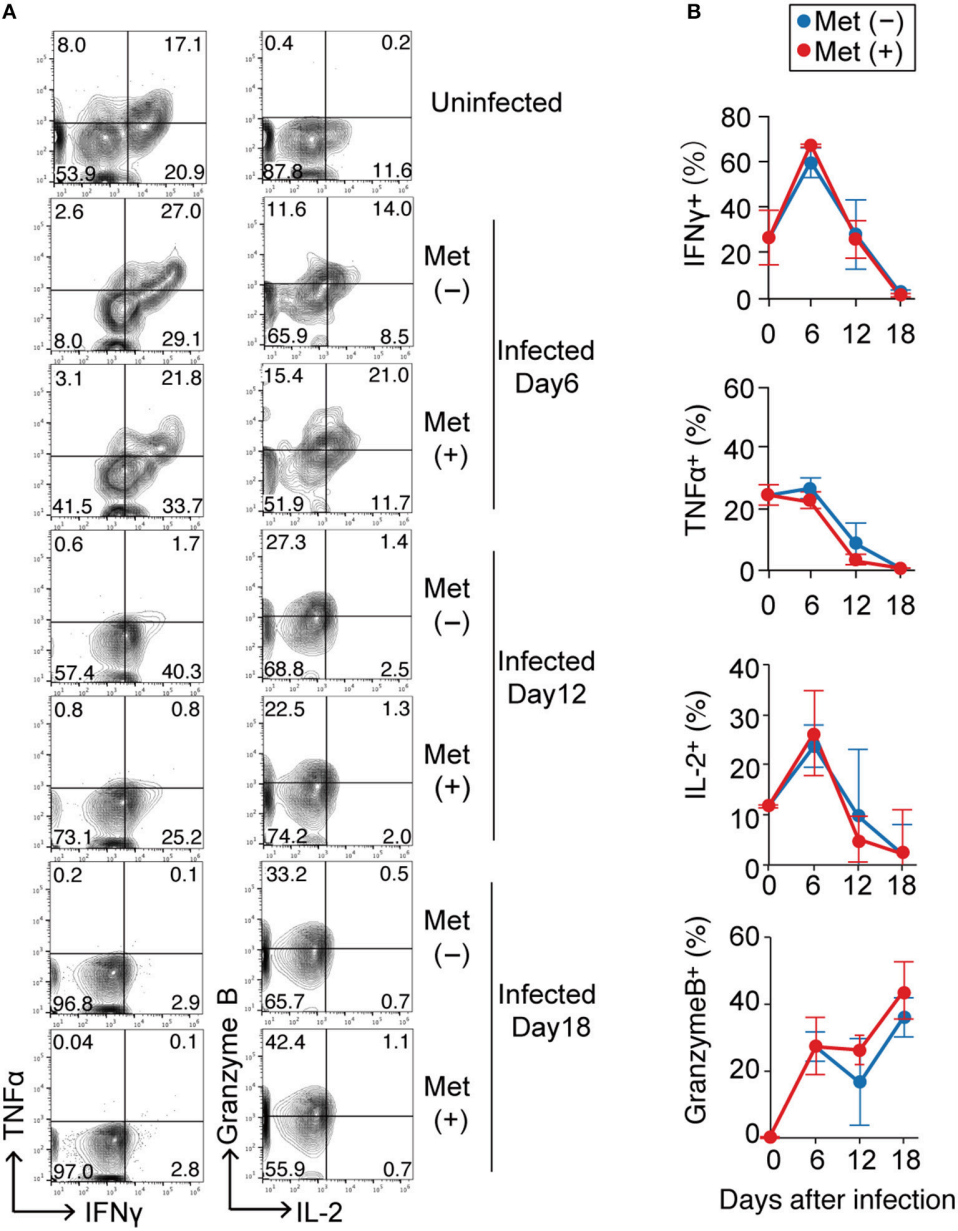
Cytokine production by γδ T cells in *Plasmodium yoelii*-infected mice treated with metformin. B6 mice were infected with *P. yoelii* 17XNL and were treated with metformin (Met^+^) or not (Met^−^), as indicated. Splenocytes from uninfected and infected mice at 6, 12, or 18 d post-infection were stimulated with PMA and ionomycin for 4 h, surfaced stained for CD3 and TCRγδ, and intracellularly stained for IFNγ/TNFα or IL-2/Granzyme B. Representative plots of these molecules on CD3^+^ TCRγδ^+^ cells **(A)** and the proportions of cytokine-positive cells (%) in metformin-treated (red) and untreated (blue) mice **(B)** are shown. Each time point represents three mice that were analyzed. Statistical analysis was performed using the unpaired *t*-test with Welch's correction. No significant difference was observed between the metformin-treated and untreated mice.

To examine the effect of metformin on γδ T cell function, we evaluated the production of cytokines and granzyme B in γδ T cells at 6, 12, and 18 d post-infection with *P. yoelii* 17XNL (Figure 6 and Figure S1). Prior to infection, 26.6 ± 11.8% and 24.4 ± 3.2% of the γδ T cells in the spleen could produce IFN-γ and TNF-α, respectively. The proportion of IFN-γ-producing γδ T cells increased at 6 d post-infection and then decreased thereafter, while that of TNF-α-producing γδ T cells continued to decrease during the infection. The γδ T cells able to produce granzyme B were not present before infection, but their numbers increased after infection. We did not detect any significant differences in the proportions of γδ T cells capable of producing cytokines and granzyme B in metformin-treated and untreated mice during the course of *Plasmodium* infection. In the αβ T cell compartment, reductions in IFN-γ- and TNF-α-producing CD4^+^ and CD8^+^ T cells were observed after 6 days of *P. yoelii* 17XNL infection. Unlike γδ T cells, production of granzyme B by CD8^+^ αβ T cells was also reduced at 6 days post-infection (Figure S5A). Serum IFN-γ levels peaked at 12 days post-infection with *P. yoelii* 17XNL, although we did not find significant difference between metformin-treated and untreated mice (Figure S5B).

### Metabolic Changes and Signaling in γδ T Cells in Metformin-Treated Mice

**Figure 7:**
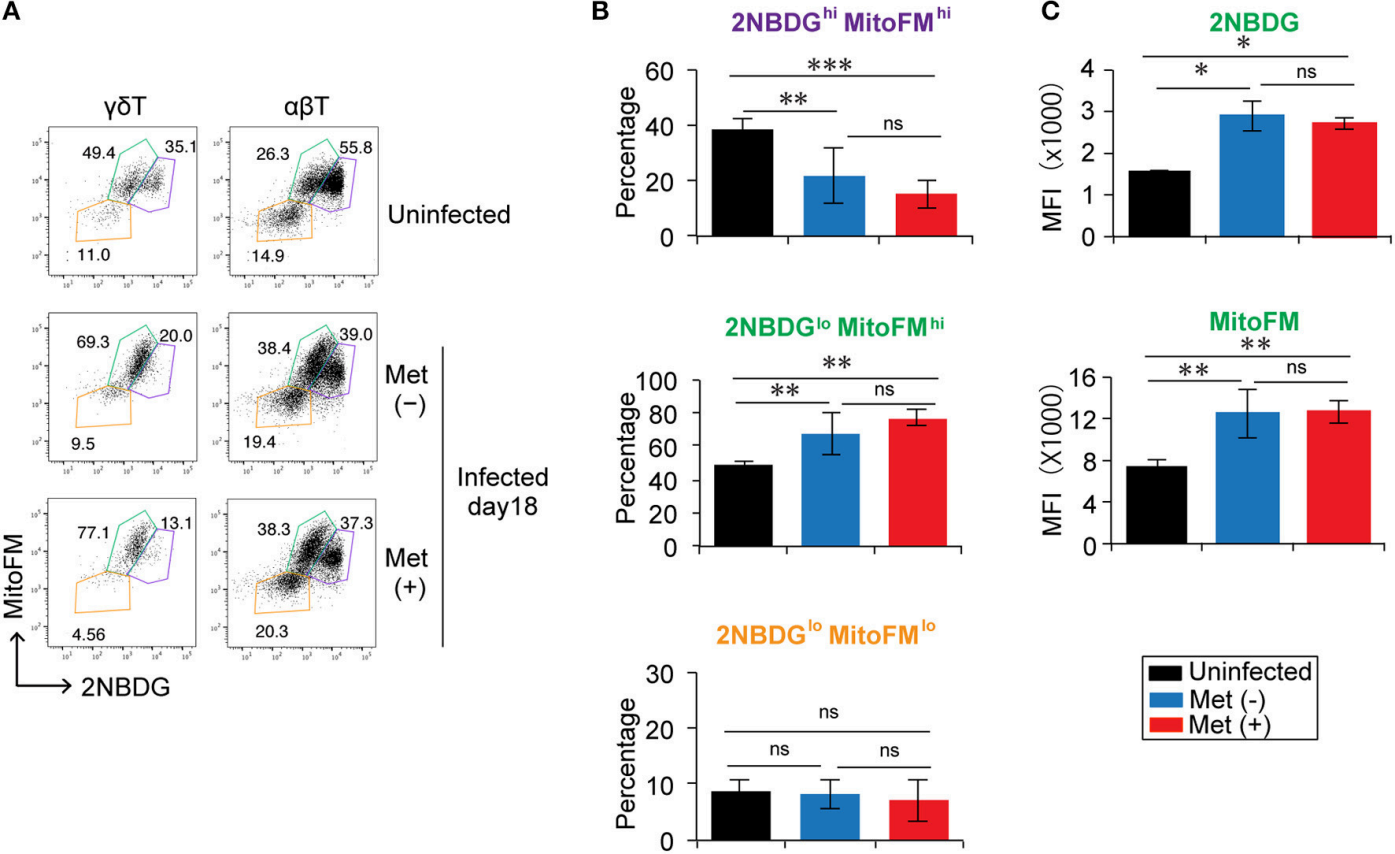
Reduction in the glucose uptake by γδ T cells in *Plasmodium yoelii* 17XNL-infected mice. B6 mice were infected with *P. yoelii* 17XNL and treated with metformin (Met^+^) or not (Met^−^), as indicated. Spleen cells from uninfected and infected mice at 18 d post-infection were incubated with 2NBDG and then stained with an anti-CD3 and anti-γδTCR mAb in combination with MitoFM. The 2NBDG vs. MitoFM profiles of γδ T cells (CD3^+^ TCRγδ^+^) and αβ T cells (CD3^+^ TCRγδ^−^) **(A)**, the proportions of 2NBDG^hi^ MitoFM ^hi^ (purple), 2NBDG^lo^ MitoFM ^hi^ (green), and 2NBDG^lo^ MitoFM^lo^ (orange) populations in CD3^+^TCRγδ^+^ cells **(B)**, and the mean fluorescence intensity (MFI) of 2NBDG^lo^ MitoFM^hi^ cells **(C)** are shown (3 mice/group). The data shown represent two independent experiments (3 or 4 mice/group) with similar results. Statistical significance was assessed using one-way ANOVA with Tukey's *post-hoc* test (^*^p < 0.05, ^**^p < 0.01, ^***^p < 0.001; ns, not significant).

Since metformin can modulate cellular metabolism through a mild, but specific inhibition of the respiratory chain complex 1 (20), we examined the metabolic function of γδ T cells in metformin-treated mice. Glucose uptake was monitored using 2NBDG, and mitochondrial mass was monitored with Mito FM staining (Figure 7 and Figure S1). The γδ T cells and αβ T cells were each divided into three subgroups of cells using 2NBDG^hi^ Mito^hi^, 2NBDG^lo^ Mito^hi^, and 2NBDG^lo^ Mito^lo^ staining as markers (Figures 7A,B). The 2NBDG^hi^ Mito^hi^ T cells were primarily naïve T cells with a CD62L^hi^ CD44^lo^ phenotype and were the primary γδ T cells in uninfected mice. The 2NBDG^lo^ Mito^hi^ cells consisted of both naïve and activated T-cells, whereas the 2NBDG^lo^ Mito^lo^ cells were a minor population that was mainly a CD62L^lo^-activated population (Figure S6). In the infected mice, the proportion of 2NBDG^hi^ Mito^hi^ cells decreased and the 2NBDG^lo^ Mito^hi^ cells became the major population of γδ T cells. The levels of 2NBDG and MitoFM in the 2NBDG^lo^ Mito^hi^ γδ T cells of the infected mice were higher compared to those in naïve mice, suggesting that glucose uptake and mitochondrial mass increased in activated γδ T cells of the infected mice (Figures 7A,C). However, we did not observe any significant differences in 2NBDG or MitoFM levels between the γδ T cells from metformin-treated and untreated mice.

**Figure 8:**
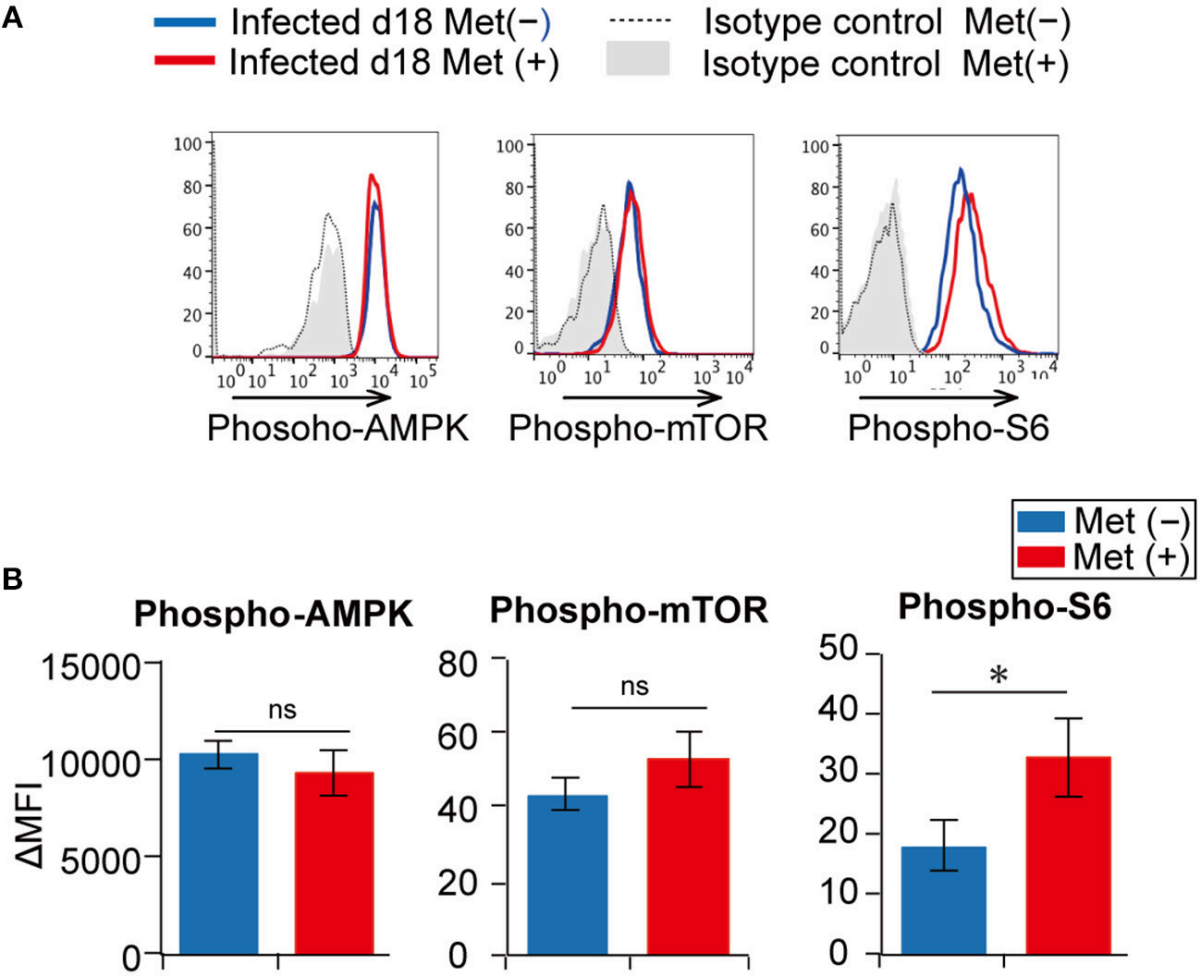
Activation of the AMPK and mTOR pathways in γδ T cells from *Plasmodium yoelii* 17XNL-infected mice. B6 mice were infected with *P. yoelii* 17XNL and treated with metformin (Met^+^) or not (Met^−^), as indicated. Splenocytes from metformin-treated (red) and untreated (blue) mice were surface stained for CD3, TCRβ, and TCRγδ, and intracellularly stained for phospho-AMPK, phospho-mTOR or phospho-S6 **(A)**. Isotype controls for metformin-treated (gray shadow) and untreated mice (dotted line) are shown. Differences in mean fluorescent intensities (ΔMFIs) between experimental samples and isotype controls in metformin-treated (red) and untreated (blue) mice are summarized **(B)**. The data shown represent two independent experiments (3 mice/group) with similar results. Statistical significance was assessed using the unpaired *t*-test with Welch's correction (^*^p < 0.05; ns, not significant).

Since metformin is reported to activate AMPK and inhibit the mTOR pathway (20, 25), we next examined AMPK activity and the mTOR pathway in γδ T cells (Figures 8A,B, Figure S1). However, we did not detect differences in the levels of AMPK and mTOR phosphorylation between metformin-treated and untreated mice by flow cytometry. Increased levels of phospho-S6, which is a downstream molecule of the mTOR pathway, was observed in the metformin-treated mice compared with that in the untreated mice, but the difference was modest. Thus, the effects of metformin on AMPK-mTOR pathway in γδ T cells appeared to be mild.

### Improved Protection in Metformin-Treated Mice Was Independent of γδT Cells

**Figure 9:**
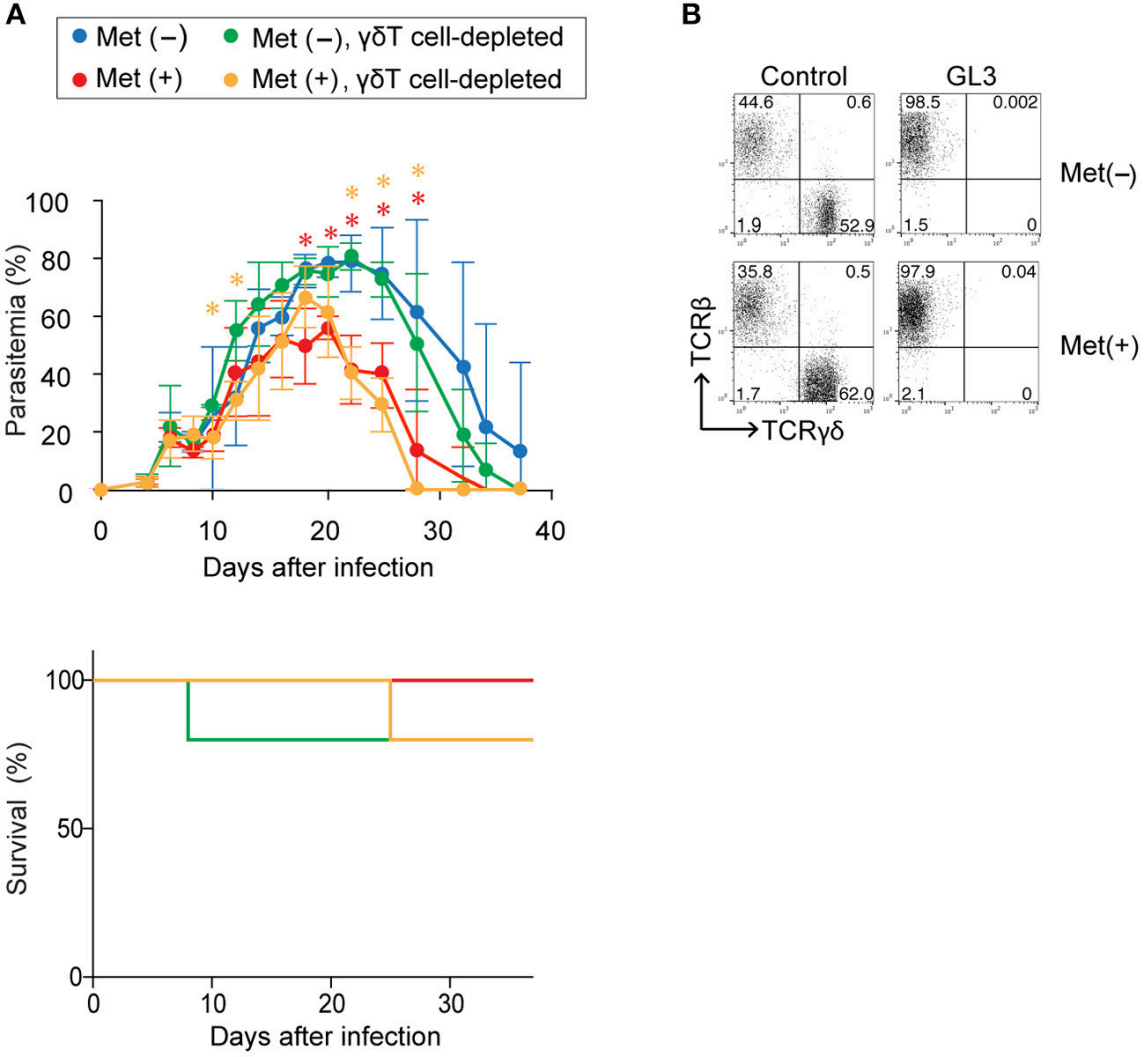
The role of γδ T cells in enhanced protection against *Plasmodium yoelii* 17XNL by metformin. B6 mice were infected with *P. yoelii* 17XNL and treated with metformin (Met^+^; red, orange) or not treated (Met^−^; blue, green). The mice were injected with an anti-γδTCR mAb (500 μg; orange, green) or PBS (red, blue) on day−1, 0, or 1 relative to infection and twice a week following infection. **(A)** Parasitemia and survival was monitored every few days. The data shown represent two independent experiments (4–5 mice/group) with similar results. Statistical significance in the differences between the metformin-treated and untreated mice among γδ T cell-sufficient (red asterisk) and γδ T cell-depleted mice (orange asterisk) was assessed by the unpaired *t*-test with Welch's correction (^*^p < 0.05). **(B)** At 40 d post-infection, splenocytes of mice treated (GL3) or not treated (control) with an anti-γδTCR mAb were stained for CD3, TCRγδ, and TCRβ expression to confirm the depletion. Representative plots of the CD3^+^-gated population are shown.

To examine whether the increased number of γδ T cells contributed to the improved clearance of parasites in metformin-treated mice, we depleted γδ T cells *in vivo* by treating the mice with an anti-TCRγδ mAb prior to infection with *P. yoelii* 17XNL (Figure 9). In mice treated with the anti-γδ TCR mAb, the population of γδ T cells was almost completely depleted (Figure 9B). The depletion of γδ T cells did not affect the levels of parasitemia in either the metformin-treated or untreated mice, and reduced parasitemia was observed irrespective of the presence or absence of γδ T cells. We therefore concluded that the improved clearance of parasites in the metformin-treated mice was independent of γδ T cells.

## Discussion

γδ T cells are unique cellular subsets of T cells that express rearranged TCR γδ and have distinct recognition properties, distributions, and functions from those of T cells expressing TCR αβ (44). Many of the γδ T cells are distributed in the front line of defense against infection, such as in skin epithelial tissue and the gastrointestinal tract, and they respond rapidly to changes in their environment and play critical roles in maintaining homeostasis in these tissues (45). Results from the current study demonstrated that γδ T cells expanded during the later phase of infection with *P. yoelii* 17XNL, which was consistent with previous studies (13–16). The expansion was strongly promoted by the administration of metformin, a drug commonly used for treating type-II diabetes. This increase in the number of γδ T cells occurred primarily in cells expressing Vγ1 or Vγ2, which localized to the red pulp of the spleen, and we suggest that it was likely mediated by increased proliferation rather than from reduced apoptosis. The selective expansion of γδ T cells suggested that TCR signaling played a role in the expansion of these cells. The expansion of Vγ1^+^ γδ T cells in mice infected with the *P. berghei* XAT strain was reported previously (46) and suggests a recognition of malaria antigens by the Vγ1 TCR. The nature of the *Plasmodium* antigens recognized by γδ T cells was not determined. We think that it is likely that metformin enhanced proliferation of γδ T cells that were activated in response to *P. yoelii* antigens.

Metformin inhibits respiratory-chain complex 1 in mitochondria and, thus, modulates the metabolic function of cells in a primarily AMPK-dependent manner (20, 25, 47). However, we were unable to detect changes in the phosphorylation of AMPK or mTOR in γδ T cells from mice treated with metformin by flow cytometry, even though the effect of metformin on the activation of AMPK were previously confirmed (28). This result suggests that the effect of metformin on AMPK activation was modest at best under the experimental conditions used in our study. However, we did detect upregulated phosphorylation of S6, a downstream target of mTOR. The level of mTOR phosphorylation was modest when compared with that observed in CD8^+^ αβT cells following TCR-mediated activation (48). Thus, we suspect that the mTOR pathway might be upregulated by metformin treatment, even though the increase in mTOR phosphorylation was undetectable by flow cytometry using our conditions. This scenario contradicts the general understanding of metformin activity on AMPK activation, which inhibits the mTOR pathway. An alternative possibility is that the upregulation of S6 phosphorylation occurred independently of mTOR activation. Further study is needed to determine the molecular signaling events that occur in γδ T cells leading to their expansion in *P. yoelii*-infected metformin-treated mice. In either case, our study showed that metformin-treatment resulted in an expansion of γδ T cells during the late phase of the infection with malaria parasites, while the number of αβT cells in the spleen remained low, suggesting that the effect of metformin on γδ T cells was distinct from that on αβ T cells.

It is known that γδ T cells secrete cytokines such as IFN-γ and TNF-α and produce granzymes, which help protect against pathogen invasion and also help maintain tissue homeostasis (42, 44, 49). Accordingly, γδ T cells upregulated the production of IFN-γ and IL-2 during the early phase of *P. yoelii* infection at a time when their numbers remained low. However, their ability to produce cytokines continued to decrease after 6 d post-infection and was almost lost by 18 d post-infection, at a point when the number of γδ T cells had clearly increased. In contrast, their ability to produce granzyme B continuously increased. These γδ T cells expressed the inhibitory receptors LAG-3, PD-1, and TIM-3, which implied that these cells entered an exhausted state as previously described (46). Metformin treatment did not affect the expression of these inhibitory receptors or the functions of the γδ T cells during infection. Taken together, these data suggest that γδ T cells increased in number during the late phase of *P. yoelii* infection but were functionally impaired due to being in an exhausted state. Therefore, these γδ T cells may not have been able to participate in protective immune responses against infection. In fact, the depletion of γδ T cells did not affect the levels of parasitemia in either the metformin-treated or untreated mice during the course of infection with *P. yoelii*.

Therefore, the question remains why treating mice with metformin reduced the levels of parasitemia. One possibility is that metformin directly inhibited the growth of *Plasmodium* parasites *in vivo*. Since metformin is a derivative of the biguanide drugs, whose anti-*Plasmodium* action was previously shown, we are unable to exclude this possibility. An alternative, but not mutually exclusive, possibility is that metformin acted on other immune system cells such as αβ T cells and B cells in the infected mice. In this study, we observed slight, but significant effects of metformin on the serum levels of anti-parasite IgG, the number of dendritic cells in the spleen on day 12 of the infection (Figure S3), and the proportion of CD62L^hi^ CD44^hi^ αβ T cells on day 18 of the infection (Figure S4). In addition, our preliminary data suggested that metformin treatment did not reduce parasitemia in Rag-2 gene-knockout mice, which lack an adaptive immune system (data not shown). Therefore, we think that the effect of metformin on reducing parasitemia depends on the host adaptive immune system, which enhances protective immunity. Further study is required to determine the mechanisms underlying the reduction of parasitemia in metformin-treated mice during *P. yoelii* infection.

In humans, γδ T cells recognize *Plasmodium* antigens and expand in a manner dependent on CD4^+^ T cells or cytokines, including IL-2, IL-4, and IL-15 (50). These γδ T cells are cytolytic to *Plasmodium* parasites and produce cytokines, and could play both protective and pathogenic roles in patients infected with *P. falciparum* (8, 9). Since metformin is used as an anti-diabetic drug in malaria-endemic regions, this drug may enhance the increase of γδ T cells in patients infected with *Plasmodium* parasites. Since these γδ T cells are functional, they might affect the clinical outcome of the infection in either positive or negative manner. Therefore, the findings of this study suggest the importance of monitoring malaria disease progression in endemic regions when patients with diabetes are treated with metformin.

The γδ T cells represent a unique T cell subset whose antigen recognition and functions are not clearly understood. These cells expand during *Plasmodium* infection and play critical protective functions by regulating both innate and adaptive immune responses (16, 49). We showed that metformin promoted the expansion of γδ T cells, but not αβ T cells, during the later phase of *Plasmodium* infection, highlighting the fact that the proliferation of γδ T cells may be regulated in a manner metabolically distinct from that of αβT cells. Further study on the metabolic function of γδ T cells and its effects on their function and clonal expansion may reveal novel strategies in enhancing protective immunity and promoting memory immune responses.

## Author Contributions

MM designed and performed the experiments, interpreted the results, and wrote the manuscript. GB performed the experiments. DK and MA provided technical advice. HU provided conceptual advice. KY designed the experiments, interpreted the results, wrote the manuscript, and supervised this study.

### Conflict of Interest Statement

The authors declare that the research was conducted in the absence of any commercial or financial relationships that could be construed as a potential conflict of interest.

## Abbreviations

2NBDG, 2-(N-(7-nitrobenz-2-oxa-1: 3-diazol-4-yl) amino)-2-deoxyglucose
7-AAD: 7-amino-actinomycin D
AMP: adenosine monophosphate
AMPK: AMP-activated protein kinase
APC: allophycocyanin
ATP: adenosine triphosphate
B6: C57BL/6
BrdU: bromodeoxyuridine
Cy7: cyanine 7
FITC: fluorescein isothiocyanate
HIF: hypoxia-inducible factor
IFN: interferon
mAb: monoclonal antibody
M-CSF: macrophage colony-stimulating factor
MFI: mean fluorescence intensity
mTOR: mammalian target of rapamycin
ns: not significant
PE: phycoerythrin
PMA: phorbol 12-myristate 13-acetate
SEM: standard error of the mean
TCR: T-cell receptor
TIL: tumor-infiltrating T cell
TNF: tumor necrosis factor
Treg: regulatory T cell
WHO: World Health Organization.
