# Supplementary material for: Metformin Promotes the Protection of Mice Infected With Plasmodium yoelii Independently of γδ T Cell Expansion
Source: Front Immunol. 2018 Dec 13;9:2942. doi: 10.3389/fimmu.2018.02942 (PMC6300485; doi:10.3389/fimmu.2018.02942)
Supplement: Supplementary file 1 [file Presentation_1.pdf]

Supplementary Material

Metformin Promotes the Protection of Mice Infected with *Plasmodium yoelii* Independently of γδ T Cell Expansion

Mana Miyakoda, Ganchimeg Bayarsaikhan, Daisuke Kimura, Masoud Akbari,

Heiichiro Udono and Katsuyuki Yui*

*** Correspondence:** Katsuyuki Yui, M.D., Ph.D.: katsu@nagasaki-u.ac.jp

1 Supplementary Figures

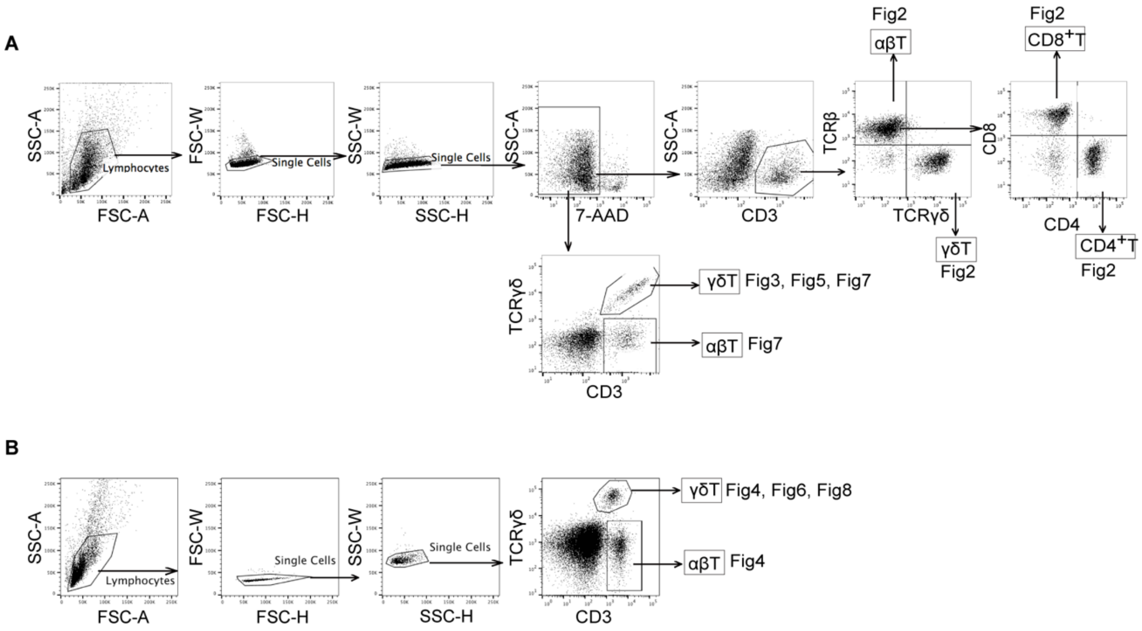

Figure S1 | Gating strategy used for flow cytometry analysis. (A) Spleen cells were stained with mAbs against CD3, TCRβ, TCRγδ, and other markers, and 7-AAD, which was used to exclude dead cells. Gating strategies for αβ T cells, γδ T cells, $CD4^+$ T cells, and $CD8^+$ T cells used to analyze their relative proportions (Fig. 2), their expression of cell-surface molecules (Fig. 3, 5), and their metabolic status (Fig. 7). **(B)** Gating strategy used with γδT cells to analyze BrdU incorporation (Fig. 4), apoptosis (Fig. 4), and intracellular staining (Fig. 6, 8).

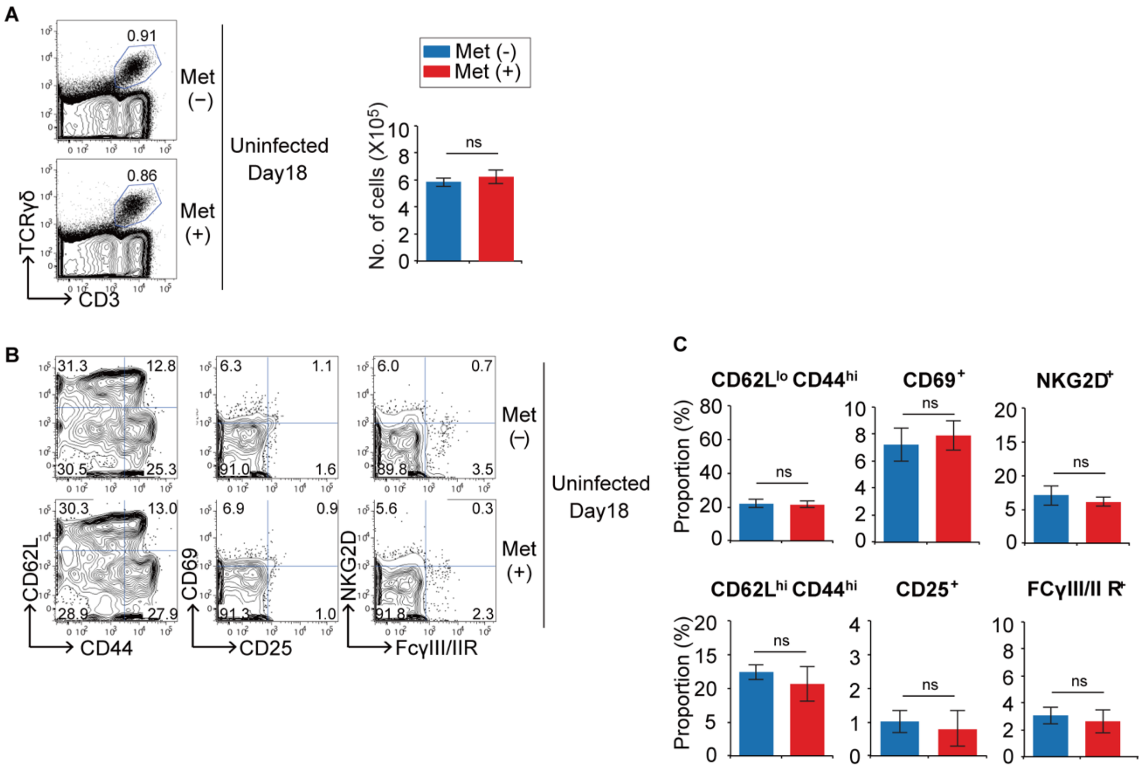

Figure S2 | The effect of metformin on γδT cells in uninfected mice. B6 mice received (red) or did not receive (blue) metformin in drinking water for 18 days (3 mice/group). **(A)** Splenocytes were stained for CD3 and TCRγδ expression, and analyzed by flow cytometry (left). The numbers in the plots indicate the proportions (%) of the cells. Total numbers of γδ T cells were calculated by multiplying the total splenocyte numbers by the proportions of γδ T cells (right). **(B, C)** Splenocytes were stained for CD3, γδTCR as well as CD62L vs. CD44, CD69 vs. CD25 and NKG2D vs. FcγIII/II R, and analyzed by flow cytometry. Representative plots of $CD3^+$ $γδTCR^+$-gated cells **(B)** and their proportions in $CD3^+$ $γδTCR^+$ cells are shown **(C)**. The numbers shown in each quadrant represent their relative proportions. Statistical significance was assessed using unpaired t-test with Welch's correction. *$p < 0.05$; ns, not significant.

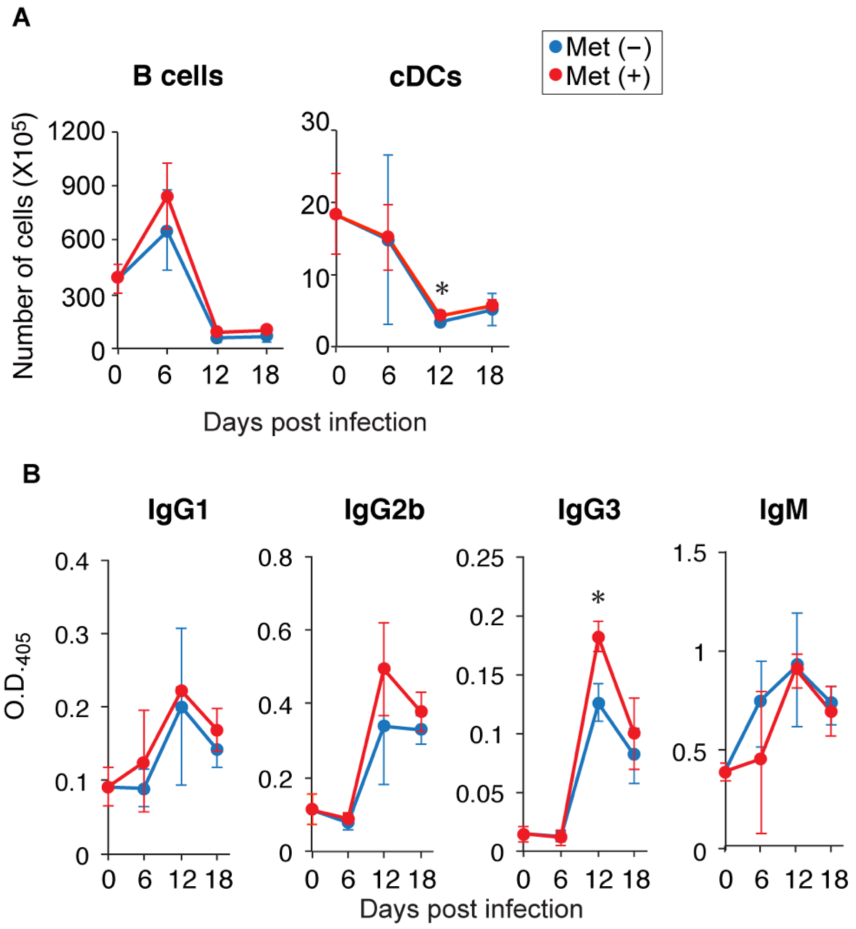

Figure S3 | The effect of metformin on B cells, dendritic cells, and antibody production in *P. yoelii*-infected mice. B6 mice were infected with *P. yoelii* 17XNL and received (Met^{+}) or did not receive (Met^{-}) metformin for 18 days, as described in Fig. 1 (3 mice/group). Mice were sacrificed at 0, 6, 12 and 18 days post-infection, and splenocyte and serum were collected. **(A)** Splenocytes from the infected mice were stained for CD19 vs. B220 or CD11c vs. MHC class II. The numbers of B cells (CD19^{+} B220^{+}) and conventional dendritic cells (CD11c^{+} MHC class II^{+}) in the spleens were calculated. **(B)** The level of serum antibodies in each mouse was determined by ELISA. Plates coated with *P. yoelii* extract were added with serum at a 1: 20 dilution, and specific antibodies were detected with biotin-conjugated anti-mouse IgG1, IgG2b, IgG3, or IgM antibodies. Statistical analysis was performed using unpaired t-test with Welch's correction. *$p < 0.05$

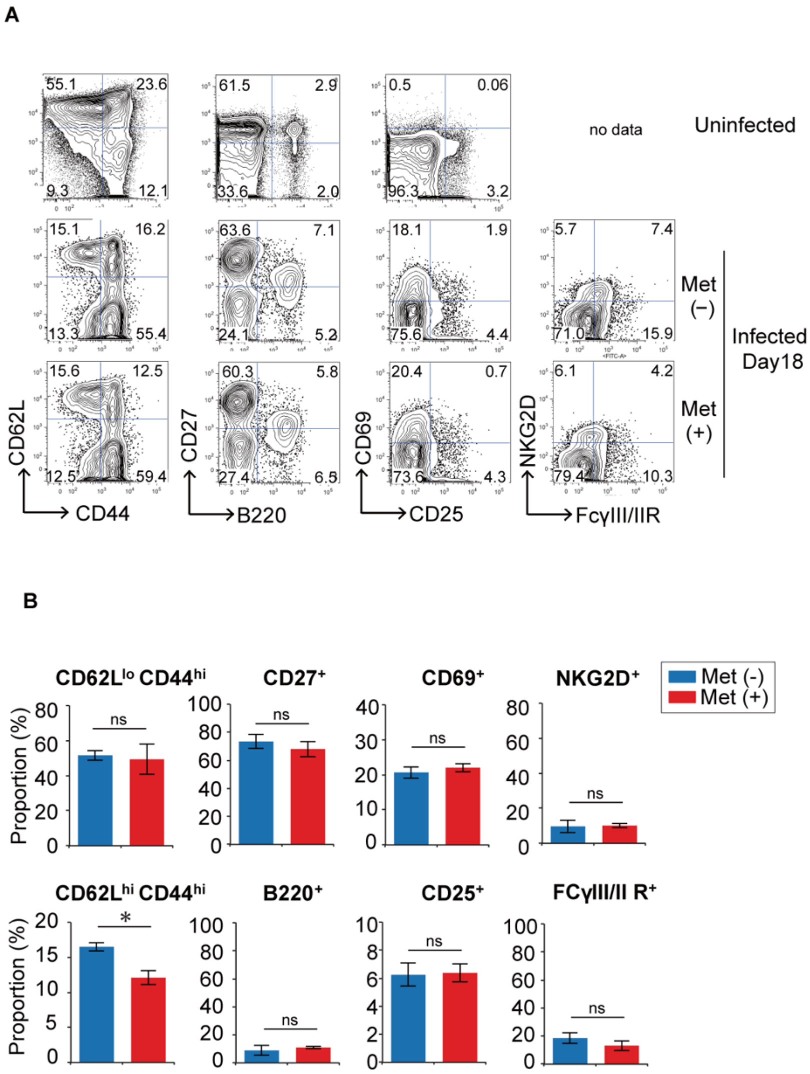

Figure S4 | The effect of metformin on the phenotype of αβT cells in *P. yoelii*-infected mice. Uninfected B6 mice or B6 mice infected with *P. yoelii* 17XNL, which received (Met^+) or did not receive (Met^-) metformin for 18 days, as described in Fig. 1 (3 mice/group). Splenocytes from uninfected and metformin-treated (Met^+) and untreated (Met^-) infected mice were stained for CD3, γδTCR, and other cell-surface markers. Representative plots of these molecules on αβ T ($CD3^+$ $γδTCR^-$) cells **(A)** and the proportions of these populations in αβ T cells are shown **(B)**. Statistical significance was assessed using the unpaired t-test with Welch's correction. *$p < 0.05$; ns, not significant.

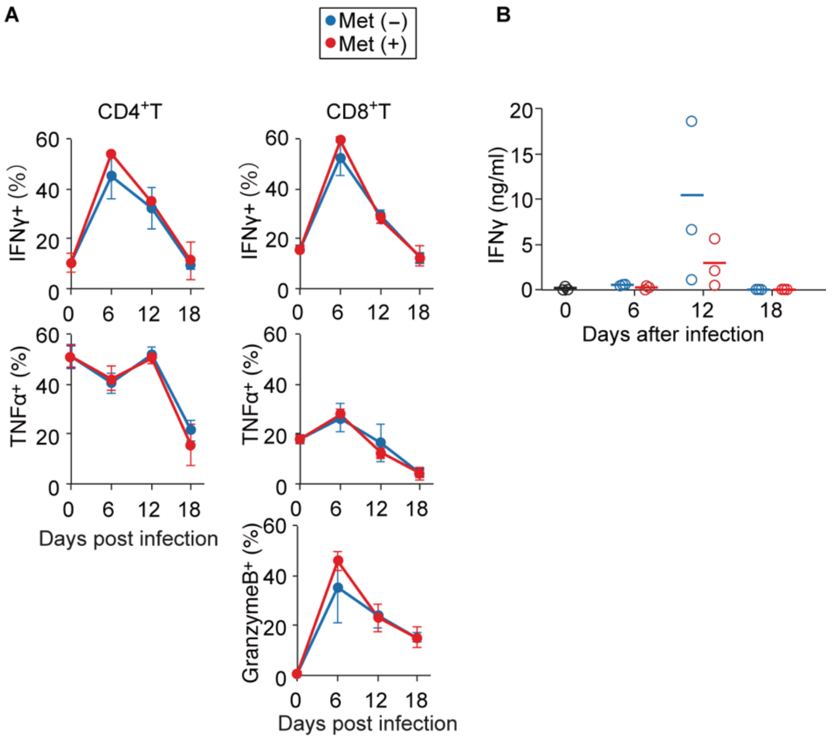

Figure S5 | Cytokine production by αβ T cells in *P. yoelii*-infected mice. B6 mice received (red) or did not receive (blue) metformin in drinking water for 18 days (3 mice/group). **(A)** Splenocytes from the infected mice were stimulated with PMA and ionomycin for 4 h; surfaced stained for CD3, CD4, and CD8; and intracellularly stained for IFN-γ, TNFα, or Granzyme B. **(B)** Serum was collected 18 days after infection, and the levels of IFN-γ were determined by ELISA. Statistical analysis was performed using the unpaired t test. No significant difference was observed between the metformin-treated and untreated mice.

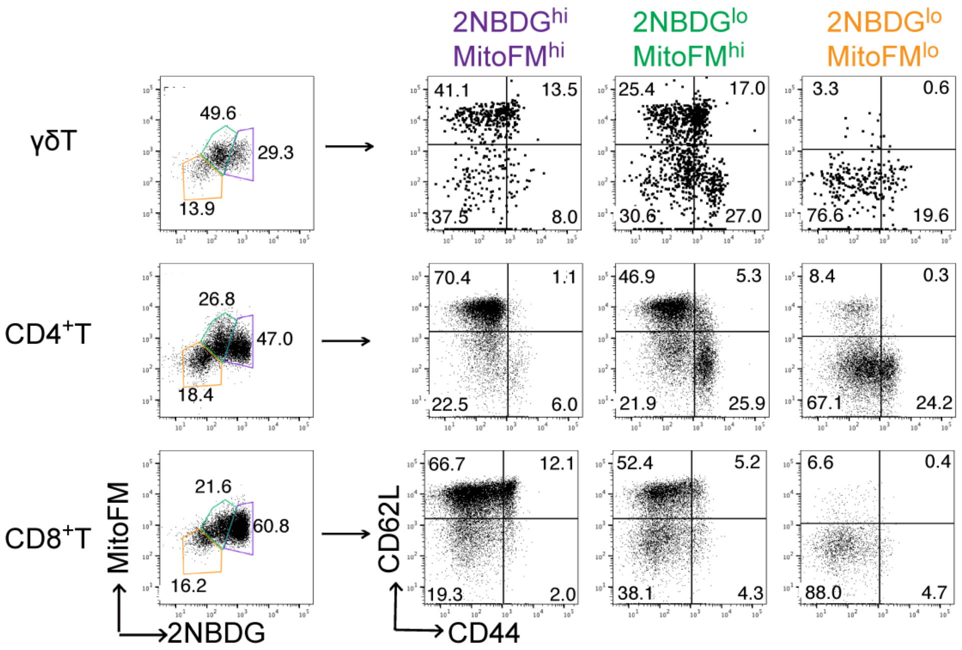

Figure S6 | Spleen cells from uninfected mice were stained for CD3, TCRγδ, CD4, CD8, CD62L, and CD44 in combination with 2NBDG and MitoFM. CD62L vs. CD44 profiles of the 2NBDG^{hi} MitoFM^{hi} (purple), 2NBDG^{lo} MitoFM^{hi} (green), and 2NBDG^{lo} MitoFM^{lo} (orange) subpopulations are shown. Numbers shown by the gating (left) and in the quadrants (right) indicate the proportions of cells. The data shown represent staining of spleen cells from 4 mice with similar results.

2 Supplementary materials and methods

ELISA

The levels of anti-*P. yoelii* IgG1, IgG2b, IgG3, and IgM antibodies were determined by ELISA. Briefly, ELISA plates were coated with freeze–thaw lysate of *P. yoelii*-infected red blood cells (8×10^6 infected RBC/ well) in PBS overnight at 4℃, and blocked with PBS containing 10% FCS for 30 minutes at room temperature. After washing plates, serum was added at 1:20 dilution and incubated 2 hrs at room temperature. Plates were washed, incubated with biotin-conjugated rabbit anti-mouse IgG1, IgG2b, or IgM antibodies (ZyMED, San Francisco, CA, USA) or biotin-conjugated rat anti-mouse IgG3 mAb (Pharmingen) at 1:1000 dilution, washed, and incubated with alkaline phosphatase-conjugated streptavidin (Jackson ImmunoResearch, West Grove, PA, USA) at 1:1000 dilution. After washing, 4-Nitrophenyl phosphate disodium salt hexahydrate (Sigma-Aldrich, MO, USA) solution was added to each well. The optical density at 405 nm was determined using a Microplate Reader (Bio-Lad, Hercules, CA, USA).

The level of IFN-γ in serum were determine by a sandwich ELISA according to the manufacturer's directions using anti-mouse IFN-γ mAb (R4-6A2) (e-Bioscience) for capture and biotinylated anti-mouse IFN-γ mAb (XMG1.2) for the detection. The optical density values were determined at 405 nm using a Microplate Reader. The concentrations of IFN-γ were calculated using a standard curve made by a serial dilution of the known concentrations of recombinant mouse IFN-γ (PeproTech, NJ, USA).
